# Rural‐urban differences in dietary intake across pregnancy trimesters: A multisite prospective cohort study

**DOI:** 10.1111/jrh.70085

**Published:** 2025-10-14

**Authors:** Alex H. Crisp, Bethany Barone Gibbs, Jacob B. Gallagher, Katrina L. Wilhite, Angela C. B. Trude, Treah Haggerty, Kara M. Whitaker

**Affiliations:** ^1^ Department of Health, Sport, and Human Physiology University of Iowa Iowa City Iowa USA; ^2^ Department of Epidemiology and Biostatistics West Virginia University Morgantown West Virginia USA; ^3^ Department of Kinesiology and HealthAffiliation Iowa State University Ames Iowa USA; ^4^ Department of Nutrition and Food Studies New York University New York City New York USA; ^5^ Department of Family Medicine West Virginia University Morganton USA

**Keywords:** food intake, maternal nutrition, rural health, socioeconomic factors

## Abstract

**Purpose:**

Poor diet during pregnancy compromises maternal–fetal health and may reflect broader environmental and structural inequities. This study investigated differences in dietary intake across pregnancy among rural and urban women in the United States and assessed whether socioeconomic status (SES) modified rural–urban differences.

**Methods:**

In this prospective study, pregnant women (*n* = 495; 22.4% rural) from three sites (Iowa, Pennsylvania, West Virginia) had dietary intake estimated via the 26‐item Dietary Screener Questionnaire (DSQ) during each trimester. Rural was defined as Rural–Urban Commuting Area (RUCA) code ≥ 4. A SES score was derived using Principal Component Analysis of education, annual household income, and insurance type. Adjusted robust linear mixed‐effects models (controlled for site, age, minority status, pre‐pregnancy BMI) compared dietary intakes between rural and urban participants, with trimester and SES interactions.

**Findings:**

Compared to their urban counterparts, rural participants had higher predicted intakes of added sugars from sugar‐sweetened beverages (SSBs) in the first (0.61 tsp eq/day; 95% CI: [0.04, 1.18]) and second trimesters (0.62 tsp eq/day [0.05, 1.21]), and less fiber across all trimesters (ranging from –0.90 g/day [–1.7, –0.1] to –1.2 g/day [–2.0, –0.3]). Women in the high‐SES urban group had higher intakes of fiber and calcium, and lower intakes of SSBs compared to their low‐SES counterparts in both rural and urban settings.

**Conclusions:**

Although rurality was associated with greater SSBs and lower fiber intake, differences were modest. Low‐SES was related to a poorer diet regardless of geography, highlighting the need for targeted interventions for both rural and urban low‐SES pregnant women.

## INTRODUCTION

Poor dietary habits are a leading risk factor for mortality from noncommunicable diseases^1^, ^2^ and impose a significant economic burden, costing an estimated $50.4 billion annually in cardiometabolic disease‐related expenses in the United States alone.^3^ Adequate nutrition is essential throughout life, but it becomes particularly critical during pregnancy to support optimal fetal development and maternal health.^4^, ^5^

Although the nutritional requirements of women increase during gestation, these needs vary across pregnancy,^6^ with dietary patterns shifting due to physiological changes, including nausea and food aversions in early pregnancy and increased caloric demands in later pregnancy.^7^ Unfortunately, the majority of pregnant women fail to meet the Dietary Guidelines for Americans,^8^, ^9^ highlighting an important public health issue that warrants further investigation into upstream factors that contribute to poor dietary intake across pregnancy trimesters.

In the United States, approximately 20% of the population resides in rural areas.^10^ Those who live in rural areas often experience geographic isolation, limited availability of supermarkets or grocery stores, long travel distances, and higher costs, which can restrict their access to affordable, nutritious food.^11^ Moreover, even when food outlets are available, they are typically convenience stores, which offer limited healthy options.^11^ These identified barriers may contribute to the higher observed rates of adverse health outcomes in rural pregnant populations, including lower birth weight and higher odds of small‐for‐gestational‐age births.^12^

The challenges associated with living in rural areas are further compounded by socioeconomic status (SES). The American Heart Association's Presidential Advisory on Rural Health reports that rural populations are heterogeneous and that disparities in cardiovascular health are closely related to social determinants of health.^10^ Socioeconomic factors such as household income, educational attainment, unemployment, and transportation barriers influence access to essential resources, including affordable fresh food options and healthcare assistance, thereby increasing vulnerability to poor dietary patterns and adverse health outcomes in rural populations.^10^ Therefore, some of the health disparities observed between rural and urban populations may reflect underlying socioeconomic inequalities rather than geographic location alone.

These structural and economic constraints highlight the importance of considering how rurality and SES intersect. While healthy eating may play a crucial role in addressing maternal and fetal health disparities in rural communities, few studies^13^, ^14^ have compared dietary intakes between rural and urban pregnant women, and none of them were conducted in the United States. Moreover, most studies including pregnant women capture dietary intake at a single time point, failing to account for trimester changes that may influence dietary behavior.^15^ This represents a critical gap in understanding of how geographic and socioeconomic barriers may disproportionately affect rural populations. Addressing these gaps is essential for developing targeted public health policies that consider geographic contexts and inform interventions aimed at improving maternal and neonatal health outcomes, particularly for underserved populations.

This study aimed to fill this gap by comparing dietary intake between pregnant women residing in rural and urban areas across each trimester. A secondary objective was to assess whether SES modified rural–urban differences. We hypothesized that rural pregnant women would have lower diet quality, characterized by reduced intake of foods rich in fiber and whole grains, such as legumes, vegetables, and fruits, and greater intake of added sugars, with SES amplifying these dietary differences.

## METHODS

### Study design

This study was part of the Pregnancy 24/7 prospective cohort, conducted at three university sites in the United States (Iowa City, Iowa; Pittsburgh, Pennsylvania; and Morgantown, West Virginia). The report adheres to the Strengthening the Reporting of Observational Studies in Epidemiology—Nutritional Epidemiology (STROBE‐nut) guidelines.^16^ The protocol is registered at ClinicalTrials.gov (NCT04749849), and details on the study protocol have been published.^17^ Participant enrollment and follow‐up were conducted between February 2021 and February 2025, with measures collected in person or remotely during three scheduled visits corresponding to the first (10–12 weeks), second (20–22 weeks), and third (32–34 weeks) trimesters of pregnancy. All participants provided written informed consent, with the University of Iowa serving as the single Institutional Review Board (number 202002630).

### Participants and recruitment

Women were eligible if they were aged 18–45 years with a gestational age of <13 weeks. Exclusion criteria included use of antihypertensive or antidiabetic medications, severe mobility conditions that limit physical activity, undergoing treatment for sleep disorders, or other serious medical conditions.

Recruitment at each university‐affiliated site involved multiple strategies, including mass e‐mails, clinical research registries, and printed and digital advertisements in clinical and community settings. To enhance rural representation, purposive outreach was conducted at the Iowa site to identify and engage individuals residing in rural areas. The West Virginia site achieved rural enrollment by partnering with multiple West Virginia University Medicine clinics providing prenatal care, including some in rural locations. Approaches to increase rural recruitment were not feasible at the Pittsburgh site due to its predominantly urban composition. To reduce barriers to participation, all sites offered fully remote enrollment and follow‐up visits, which were particularly important for women experiencing geographic or transportation challenges.

A previous power calculation based on preliminary data from the Pregnancy 24/7 cohort (*n* = 350, *α* = 0.05, power = 0.80) indicated that the study had sufficient power to detect a small effect size (Cohen's *d* = 0.212). This effect size corresponds to the ability to detect rural–urban differences as small as 1.06 tsp/day of added sugars, 0.07 oz/day of whole grains, 0.12 cups/day of dairy, 0.14 cups/day of fruits and vegetables, and 34.5 mg/day of calcium.

### Dietary intake

Dietary intake was assessed using the paper version of the self‐administered Dietary Screener Questionnaire (DSQ), developed by the National Cancer Institute.^18^ The DSQ is a brief food frequency questionnaire that asks participants to report the frequency of consumption of 26 food items over the past 30 days, selected for their relevance to key dietary components. The DSQ was included in the 2009–2010 National Health and Nutrition Examination Survey (NHANES), from which scoring algorithms were developed to predict daily intake of fruits and vegetables, dairy, added sugars, added sugars from sugar‐sweetened beverages (SSBs), whole grains, fiber, and calcium. Dietary intake estimates were derived for each trimester using a multi‐step algorithm. First, categorical frequency responses were numerically converted to estimated daily frequencies, with top‐coding procedures incorporated to address implausibly high intake values based on the 2009–2010 NHANES distribution, thereby minimizing the influence of outliers without excluding participants. These frequencies were then adjusted using portion size factors specific to general sex and age categories, as established by the DSQ scoring algorithm. Nutrient‐specific estimates were derived using calibration coefficients, and cereal‐specific intake was further refined based on reported cereal types and nutrient content. This study used the following DSQ‐derived outcomes: fruits (cup eq/day), vegetables (including legumes and excluding french fries; cup eq/day), dairy (cup eq/day), total added sugars (tsp eq/day), added sugars from SSBs (tsp eq/day), whole grains (oz eq/day), fiber (g/day), and calcium (mg/day). The full version of the Dietary Screener Questionnaire (DSQ), which includes all constituent items, response categories, and administration instructions, is publicly available from the National Cancer Institute at: https://epi.grants.cancer.gov/nhanes/dietscreen/questionnaires.html. A comprehensive description of the statistical methods employed to estimate dietary intake based on DSQ responses and validation is detailed in Thompson et al.^18^

### Rurality

Rural status was determined using the Rural–Urban Commuting Area (RUCA) codes, a classification system developed by the United States Department of Agriculture (USDA).^19^ RUCA codes categorize US census tracts based on measures of population density, urbanization, and daily commuting patterns, providing a nuanced assessment of rurality. Participants’ home addresses were geocoded and linked to the corresponding census tract to assign a RUCA code ranging from 1 (most urban) to 10 (most rural). For this analysis, RUCA codes were dichotomized: codes 1–3 were classified as urban and codes 4–10 as rural.^20^

### Socioeconomic status (SES)

A composite SES index was derived using Principal Component Analysis (PCA) applied to three variables—total annual household income (with categories ranging from “less than $50,000” to “$150,000 or more” per year), education (with categories from “high school” to “college degree”), and insurance coverage (private or public)—all self‐reported by women and collected during their first trimester. These indicators were selected due to their well‐established associations with health outcomes and social determinants of health.^21^ Prior to conducting the PCA, data suitability was assessed using the Kaiser–Meyer–Olkin measure, and Bartlett's test of sphericity was statistically significant, confirming the factorability of the correlation matrix. The PCA was performed using the FactoMineR package (version 2.11), and only the first principal component was retained, as it had an eigenvalue greater than 1 and accounted for 71.4% of the total variance. To facilitate interpretation and ensure adequate power for subgroup comparisons, the SES index was dichotomized at the sample median into “low” and “high” SES categories based on the component scores. This classification supported the study's focus on urban–rural differences in dietary intake and ensured sufficient sample sizes for statistical comparisons.

### Sociodemographic characteristics

Sociodemographic variables, including age, race (White/Caucasian, Black/African American, Asian, Others), ethnicity (Hispanic or Latin), marital status (married, married‐like relationship, separated/divorced, single/not married), insurance type (Medicaid, Medicare, private, none, don't know), educational attainment (high school, some college/associated degree, post‐college degree), and total annual household income (don't know/refused, < $50,000, $50,000–$149,999, ≥ $150,00), were self‐reported during the first‐meeting questionnaire. Race and ethnicity responses were used to construct a binary indicator of underrepresented minority status, defined as identifying with any non‐White race or reporting Hispanic/Latin ethnicity. Body mass index (BMI) was calculated from height measured during the first in‐person visit and pre‐pregnancy weight abstracted from medical charts. Height measurements were taken in duplicate, with a third measurement performed if the first two differed by more than 0.5 cm. The average of the two closest values was considered for analysis. For participants who completed remote visits, height was self‐reported.

### Statistical analysis

All analyses were conducted in R (version 4.4.3). Descriptive comparisons of participant characteristics by urban–rural residence were performed using independent t‐tests for continuous variables and chi‐square tests or Fisher's exact tests for categorical variables. To assess changes in dietary intake across pregnancy by urban–rural residence, linear mixed‐effects models with robust estimation were fitted using the robustlmm package (version 3.3‐1).^22^ The models examined the differences of time (trimester), residential setting (urban vs. rural), and their interaction on each dietary outcome derived from the DSQ, adjusting for study site, age, minority status, and pre‐pregnancy BMI as fixed effects. These covariates were selected a priori due to their established relevance as potential confounders in nutritional epidemiology. For the second set of analyses, adjusted robust linear mixed models included a two‐way (trimester x SES) and a three‐way interaction term (trimester × rurality × SES) to explore the role of SES, modeled as a binary variable (low vs. high). All models included a random intercept for participant ID to account for repeated measures across trimesters. Linear mixed models were chosen to better deal with imbalanced sample sizes between urban and rural participants. Robust estimation was applied to mitigate the influence of heteroscedasticity and outliers identified during preliminary model diagnostics using the performance package (version 0.13.0), ensuring that neither group disproportionately influences the overall estimates.

Pairwise contrasts were conducted using the emmeans package (version 1.11.0) to compare dietary intake between urban and rural participants at each trimester, as well as changes across trimesters within each group (urban and rural). Multiple comparisons were adjusted using the Holm–Bonferroni method to control the family‐wise error rate. Significant differences (two‐tailed *p* < 0.05) were reported as paired differences with 95% confidence intervals. To quantify inter‐individual variability, the adjusted intraclass correlation coefficient (ICC) was calculated using variance components from the random‐effects structure of each model.^23^ To assess the influence of the sample imbalance and consistency of findings, sensitivity analyses were performed by fitting separate, site‐specific models for Iowa and West Virginia, but not for Pittsburgh, due to its small rural sample.

## RESULTS

The Pregnancy 24/7 Study cohort enrolled 500 participants, of whom 495 provided complete dietary data for at least one trimester and were included in the final analytical sample (Figure A1). Retention rates were high across all trimester visits: 384 urban and 111 rural participants in the first trimester (total 495); 354 urban and 99 rural in the second trimester (total 453); and 349 urban and 94 rural in the third trimester (total 443). Demographic characteristics of the study sample are summarized in Table 1.

**TABLE 1:** Sociodemographic characteristics of pregnant participants by urban and rural residence, the Pregnancy 24/7 cohort study.

	Overall (*n* = 495)	Urban (*n* = 384)	Rural (*n* = 111)	*p*‐value
**Age, mean ± SD**	30.7 ± 4.5	31.0 ± 4.4	29.6 ± 5.0	0.012
**Pre‐pregnancy BMI, mean ± SD**	27.0 ± 6.6	26.6 ± 6.3	28.4 ± 7.6	0.026
**Study site, *n* (%)**				
Iowa City (Iowa)	250 (50.5)	188 (49.0)	62 (55.9)	<0.001
Pittsburgh (Pittsburgh)	124 (25.1)	121 (31.5)	3 (2.7)	
Morganton (West Virginia)	121 (24.4)	75 (19.5)	46 (41.4)	
**Race, *n* (%)**				
White/Caucasian	427 (86.3)	319 (83.1)	108 (97.3)	0.002
Black/African American	28 (5.7)	27 (7.0)	1 (0.9)	
Asian	24 (4.8)	23 (6.0)	1 (0.9)	
Multiple races	13 (2.6)	12 (3.1)	1 (0.9)	
Native Hawaiian or Other Pacific Islander	3 (0.1)	3 (0.1)	0 (0)	
**Hispanic/Latin, *n* (%)**				
No	409 (82.6)	306 (79.7)	103 (92.8)	0.002
Yes	86 (17.4)	78 (20.3)	8 (7.2)	
**Marital status, *n* (%)**				
Married	392 (79.2)	316 (82.3)	76 (68.5)	0.007
Married‐like relationship	53 (10.7)	33 (8.6)	20 (18.0)	
Separated/divorced	3 (0.1)	3 (0.1)	0 (0)	
Single/not married	47 (9.5)	32 (8.3)	15 (13.5)	
**Insurance, *n* (%)**				
Private	413 (83.4)	333 (86.7)	80 (72.1)	0.001
Public (Medicaid/Medicare)	79 (16.0)	48 (12.5)	31 (27.9)	
None/don't know	3 (0.1)	3 (0.1)	0 (0)	
**Education, *n* (%)**				
High school	51 (10.3)	32 (8.3)	19 (17.1)	<0.001
Some college or associate degree	69 (13.9)	40 (10.4)	29 (26.1)	
College degree	166 (33.5)	125 (32.6)	41 (36.9)	
Post‐college degree	209 (42.2)	187 (48.7)	22 (19.8)	
**Household income, *n* (%)**				
< $50,000	84 (17.0)	57 (14.8)	27 (24.3)	0.001
$50,000–$149,999	276 (55.8)	213 (55.5)	63 (56.8)	
≥ $150,000	121 (24.4)	106 (27.6)	15 (13.5)	
Don't know/refused	14 (2.8)	8 (2.1)	6 (5.4)	

*Note*: *p*‐values derived from independent samples *t*‐tests for continuous variables and chi‐square or Fisher's exact tests for categorical variables.

Abbreviation: BMI, body mass index.

Pregnant women residing in rural areas had a lower mean age (–1.4 years; 95% CI: –2.3, –0.4) but higher mean pre‐pregnancy BMI (1.8 kg/m^2^; 95% CI: 0.4, 3.2) compared to those in urban areas. Rural participants exhibited lower racial diversity, being predominantly non‐Hispanic White (97.3%), whereas the urban group presented higher levels of education (48.7%, post‐college degree) and household income (27.6%, ≥ $150,000). Being married was more common among urban participants (82.3% vs. 68.5%), whereas married‐like relationships (18.0% vs. 8.6%) and being single or not married (13.5% vs. 8.3%) were more frequently reported among rural residents. Although most participants reported having private health insurance, the use of public programs such as Medicaid or Medicare was more frequent in the rural group (27.9% vs. 12.5%).

Table 2 presents trimester‐specific estimates of the primary outcomes stratified by residential setting (rural vs. urban). Within‐group changes across trimesters were small; dairy intake increased from the first to the third trimester in both urban and rural participants, while total added sugars and SSB intake increased primarily among those living in urban areas, and calcium intake among participants from rural areas.

**TABLE 2:** Estimated dietary intake across pregnancy by setting (urban vs. rural) and trimester.

Dietary		Trimester				
Component	Setting	1	2	3	T2–T1	T3–T1
Fruits (cup eq/day)	Rural	0.93 [0.84, 1.01]	0.92 [0.83, 1.01]	0.96 [0.88, 1.05]	−0.01 [–0.08, 0.06]	0.03 [–0.04, 0.11]
	Urban	1.01 [0.97, 1.06]	1.03 [0.98, 1.08]	1.03 [0.98, 1.08]	0.02 [–0.02, 0.06]	0.02 [–0.02, 0.06]
	Diff	−0.08 [–0.21, 0.04]	−0.11 [–0.24, 0.02]	−0.07 [–0.20, 0.06]		
Vegetables (cup eq/day)	Rural	1.23 [1.18, 1.29]	1.27 [1.22, 1.33]	1.28 [1.22, 1.33]	0.04 [–0.00, 0.08]	0.05 [–0.00, 0.08]
	Urban	1.31 [1.28, 1.34]	1.31 [1.28, 1.34]	1.33 [1.30, 1.36]	0.00 [–0.02, 0.02]	0.02 [–0.01, 0.04]
	Diff	−0.08^*^ [–0.13, –0.02]	−0.04 [–0.09, 0.02]	−0.05 [–0.11, 0.01]		
Dairy (cup eq/day)	Rural	1.45 [1.37, 1.54]	1.56 [1.47, 1.64]	1.54 [1.45, 1.63]	0.11^*^ [0.02, 0.20]	0.09^*^ [0.00, 0.18]
	Urban	1.50 [1.45, 1.55]	1.53 [1.48, 1.58]	1.55 [1.50, 1.60]	0.03 [–0.02, 0.08]	0.05^*^ [0.00, 0.10]
	Diff	−0.05 [–0.17, 0.07]	0.03 [–0.09, 0.16]	−0.01 [–0.14, 0.12]		
Total added sugars (tsp eq/day)	Rural	14.5 [13.5, 15.1]	15.1 [14.2, 15.8]	15.2 [14.2, 15.9]	0.6 [–0.1, 1.4]	0.7 [–0.0, 1.4]
	Urban	14.0 [13.3, 14.4]	14.8 [14.1, 15.2]	14.8 [14.1, 15.2]	0.8^*^ [0.4, 1.2]	0.8^*^ [0.5, 1.2]
	Diff	0.5 [–0.46, 1.49]	0.3 [–0.63, 1.37]	0.4 [–0.65, 1.37]		
Added sugars from SSB (tsp eq/day)	Rural	5.70 [5.31, 6.09]	5.91 [5.52, 6.31]	5.75 [5.34, 6.15]	0.21 [–0.16, 0.59]	0.05 [–0.34, 0.43]
	Urban	5.09 [4.86, 5.31]	5.29 [5.06, 5.52]	5.33 [5.10, 5.56]	0.20^*^ [0.01, 0.40]	0.24^*^ [0.05, 0.45]
	Diff	0.61^*^ [0.04, 1.18]	0.62^*^ [0.05, 1.21]	0.42 [–0.17, 1.00]		
Whole grains (oz eq/day)	Rural	0.65 [0.59, 0.72]	0.68 [0.61, 0.74]	0.69 [0.62, 0.75]	0.03 [–0.05, 0.09]	0.04 [–0.04, 0.10]
	Urban	0.72 [0.69, 0.76]	0.70 [0.66, 0.73]	0.71 [0.67, 0.75]	−0.02 [–0.06, 0.01]	−0.01 [–0.05, 0.02]
	Diff	−0.07 [–0.16, 0.02]	−0.02 [–0.11, 0.07]	−0.02 [–0.12, 0.07]		
Fiber (g/day)	Rural	14.2 [13.7, 14.8]	14.3 [13.8, 14.9]	14.6 [14.0, 15.2]	0.1 [–0.4, 0.6]	0.4 [–0.1, 0.9]
	Urban	15.4 [15.1, 15.7]	15.3 [15.0, 15.7]	15.5 [15.1, 15.8]	−0.1 [–0.3, 0.2]	0.1 [–0.2, 0.4]
	Diff	−1.2^*^ [–2.0, –0.3]	−1.0^*^ [–1.8, –0.2]	−0.9^*^ [–1.7, –0.1]		
Calcium (mg/day)	Rural	868 [841, 896]	900 [872, 928]	900 [871, 929]	32^*^ [1, 63]	32^*^ [1, 64]
	Urban	911 [895, 927]	915 [899, 931]	926 [910, 942]	4 [–12, 21]	15 [–2, 32]
	Diff	−43^*^ [–83, –2]	−15 [–57, 26]	−26 [–68, 17]		

*Note*: Values are estimated marginal means [95% confidence intervals] derived from robust linear mixed‐effects models adjusted for age, minority status, pre‐pregnancy BMI, and study site. “Diff” represents the difference between rural and urban participants.

* Indicates statistical significance at *p* < 0.05.

Rural–urban comparisons revealed significant differences in specific dietary components. During the first and second trimesters, rural participants had higher added sugars from SSBs intake by 0.61–0.62 tsp eq/day (95% CI: 0.04, 1.21) compared to urban participants. In contrast, fiber intake was consistently lower in rural participants across pregnancy trimesters by 0.9–1.2 g/day (95% CI: –2.0, –0.1). Additionally, vegetable intake was significantly lower in rural participants during the first trimester (–0.08 cup eq/day; 95% CI: –0.13, –0.02), though this difference was not statistically significant in the second or third trimesters.

No significant rural–urban paired contrast differences were observed for the predicted intake of fruits, whole grains, or dairy in any trimester. In all models, the adjusted ICCs ranged from 0.61 to 0.77, indicating that a substantial proportion of the variability in dietary intake was due to inter‐individual differences rather than changes across trimesters or location. A sensitivity analysis restricted to participants from Iowa and West Virginia (Supporting Information **Table** **A1**) confirmed that rural participants had lower fiber intake and higher consumption of added sugars from SSBs compared to their urban counterparts, corroborating that rural–urban differences were consistent across sites.

An initial analysis comparing SES groups (Table 3) showed that low‐SES participants consistently had 1.29–1.49 tsp eq/day (95% CI: 0.57, 1.99) higher intake of added sugars from SSBs, 1.5–1.6 g/day (95% CI: 0.8, 2.4) lower fiber intake, and 31–58 mg/day (95% CI: –96, –4) lower calcium intake than high‐SES participants. Differences in total added sugar intake were also significant, with low‐SES participants having 1.4 tsp eq/day (95% CI: 0.5, 2.3) higher intake than high‐SES participants in early and mid‐pregnancy. For fruit intake, only the third trimester showed a significant pairwise contrast, with low‐SES participants having 0.12 cup eq/day (95% CI: –0.24, –0.01) lower intake than high‐SES participants.

**TABLE 3:** Mean estimates of dietary intake by trimester and socioeconomic status.

Dietary		Trimester				
Component	SES	1	2	3	T2–T1	T3–T1
Fruits (cup eq/day)	Low	0.96 [0.89, 1.03]	0.95 [0.88, 1.03]	0.94 [0.86, 1.01]	−0.01 [–0.07, 0.06]	−0.02 [–0.09, 0.05]
	High	1.03 [0.97, 1.08]	1.04 [0.99, 1.10]	1.06 [1.01, 1.12]	0.01 [–0.02, 0.06]	0.03 [–0.01, 0.07]
	Diff	−0.07 [–0.18, 0.04]	−0.09 [–0.20, 0.03]	−0.12^*^ [–0.24, –0.01]		
Vegetables/legumes (cup eq/day)	Low	1.27 [1.22, 1.31]	1.27 [1.22, 1.32]	1.29 [1.24, 1.34]	0.00 [–0.05, 0.05]	0.02 [–0.03, 0.07]
	High	1.31 [1.28, 1.35]	1.32 [1.29, 1.36]	1.34 [1.30, 1.37]	0.01 [–0.02, 0.04]	0.03 [–0.01, 0.07]
	Diff	−0.04 [–0.12, 0.03]	−0.05 [–0.13, 0.02]	−0.05 [–0.12, 0.03]		
Dairy (cup eq/day)	Low	1.50 [1.44, 1.57]	1.56 [1.49, 1.64]	1.50 [1.43, 1.57]	0.06 [–0.2, 0.14]	0.00 [–0.08, 0.08]
	High	1.48 [1.43, 1.54]	1.52 [1.47, 1.58]	1.56 [1.51, 1.62]	0.04 [–0.01, 0.09]	0.08^*^ [0.03, 0.13]
	Diff	0.02 [–0.09, 0.13]	0.04 [–0.07, 0.15]	−0.06 [–0.17, 0.05]		
Total added sugars (tsp eq/day)	Low	15.0 [14.4, 15.5]	15.8 [15.2, 16.4]	15.4 [15.1, 16.2]	0.8 [0.2, 1.5]	0.4 [–0.2, 1.0]
	High	13.6 [13.6, 14.3]	14.4 [14.4, 15.1]	14.6 [14.8, 15.9]	0.8 [0.4, 1.1]	1.0 [0.6, 1.4]
	Diff	1.4^*^ [0.5, 2.2]	1.4^*^ [0.5, 2.3]	0.8 [–0.2, 1.7]		
Added sugars from SSB (tsp eq/day)	Low	6.07 [5.76, 6.38]	6.44 [6.11, 6.77]	6.13 [5.80, 6.46]	0.37^*^ [0.05, 0.71]	0.06 [–0.27, 0.40]
	High	4.78 [4.54, 5.03]	4.95 [4.71, 5.20]	5.03 [4.79, 5.28]	0.17 [–0.03, 0.38]	0.25^*^ [0.05, 0.46]
	Diff	1.29^*^ [0.79, 1.79]	1.49^*^ [0.96, 1.99]	1.10^*^ [0.57, 1.62]		
Whole grains (oz eq/day)	Low	0.66 [0.61, 0.71]	0.64 [0.59, 0.70]	0.67 [0.62, 0.73]	−0.02 [–0.07, 0.04]	0.01 [–0.05, 0.07]
	High	0.73 [0.70, 0.77]	0.72 [0.68, 0.76]	0.73 [0.69, 0.77]	−0.01 [–0.05, 0.02]	0.00 [–0.04, 0.03]
	Diff	−0.07 [–0.16, 0.01]	−0.08 [–0.16, 0.01]	−0.06 [–0.14, 0.03]		
Fiber (g/day)	Low	14.2 [13.7, 14.6]	14.1 [13.6, 14.5]	14.3 [13.8, 14.7]	−0.1 [–0.6, 0.3]	0.1 [–0.4, 0.5]
	High	15.7 [15.4, 16.1]	15.7 [15.4, 16.1]	15.9 [15.5, 16.2]	0.0 [–0.2, 0.3]	0.2 [–0.1, 0.4]
	Diff	1.5^*^ [0.8, 2.2]	1.6^*^ [1.0, 2.4]	1.6^*^ [0.9, 2.4]		
Calcium (mg/day)	Low	883 [861, 906]	891 [867, 915]	881 [857, 905]	8 [–19, 35]	−2 [–30, 25]
	High	914 [896, 931]	925 [907, 943]	939 [922, 957]	11 [–6, 28]	25^*^ [9, 42]
	Diff	−31^*^ [–67, –71]	−34^*^ [–71, –4]	−58^*^ [–96, –20]		

*Note*: Values are estimated marginal means [95% confidence intervals] derived from robust linear mixed‐effects models adjusted for age, minority status, pre‐pregnancy BMI, and study site. “Diff” represents the difference between urban and rural participants.

* Indicates statistical significance at *p* < 0.05.

**FIGURE 1:**
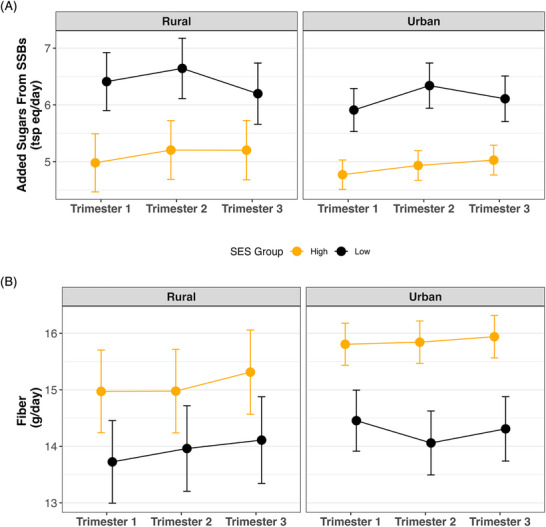
Predicted intake of (A) added sugars from sugar‐sweetened beverages (SSBs) and (B) fiber across pregnancy, stratified by socioeconomic status (SES) and residential setting. Values represent estimated marginal means with 95% confidence intervals, derived from robust linear mixed‐effects models adjusted for age, minority status, pre‐pregnancy BMI, and study site.

Concerning the intersection of rurality and SES, the analysis revealed layers of privilege that were independent of residential setting for SSBs intake. Across pregnancy, urban‐high SES participants had a SSBs intake 1.1–1.4 tsp eq/day (95% CI: –2.1, –0.04) lower than their low‐SES counterparts. Similarly, rural‐high SES participants had a SSBs intake 1.0–1.4 tsp eq/day (95% CI: –2.5, –0.01) lower than their low‐SES counterparts. Rural‐high SES participants also had a SSBs intake 0.9–1.1 tsp eq/day (95% CI: –2.1, –0.01) lower than urban‐low SES participants, while urban‐high SES participants had an even lower intake compared to rural‐low SES participants, with a difference of 1.2–1.7 tsp eq/day (95% CI: –2.6, 0.4) (Figure 1A).

In contrast, SES‐related differences in fiber intake were particularly evident in urban settings, where urban‐high SES participants consistently had a fiber intake 1.4–2.1 g/day (95% CI: 0.2, 3.3) higher than their low‐SES counterparts. Specifically, they had a fiber intake 1.4–1.8 g/day (95% CI: 0.2, 3.0) higher than urban‐low SES participants and 1.8–2.1 g/day (95% CI: 0.6, 3.3) higher than rural‐low SES participants (Figure 1B).

**FIGURE 2:**
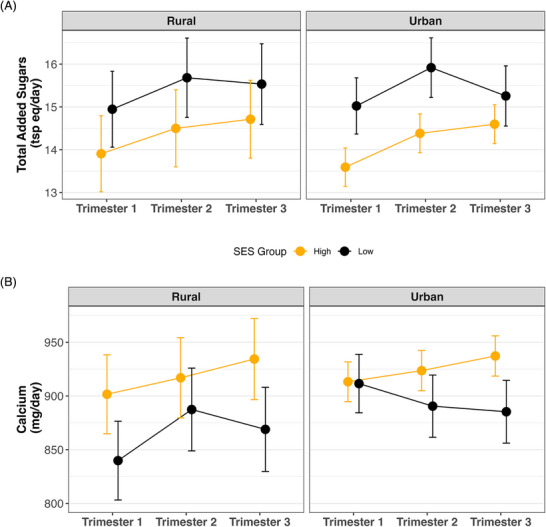
Predicted intake of (A) total added sugars and (B) calcium across pregnancy, stratified by socioeconomic status (SES) and residential setting. Values represent estimated marginal means with 95% confidence intervals, derived from robust linear mixed‐effects models adjusted for age, minority status, pre‐pregnancy BMI, and study site.

Although the primary analysis did not reveal consistent rural–urban differences in total added sugars, this intersectional analysis showed that urban high‐SES participants had a total added sugar intake 1.4–1.5 tsp eq/day (95% CI: –2.8, –0) lower than urban low‐SES participants, particularly in the first two trimesters (Figure 2A). Additionally, urban high‐SES participants had 63–73 mg/day (95% CI: 6, 132) higher calcium intake compared to rural‐low SES participants in the second and third trimesters, while differences between urban‐high and urban‐low SES participants were observed only in the third trimester (51 mg/day; 95% CI: (0.4, 103)) (Figure 2B). For other dietary components—including fruits, vegetables/legumes, dairy, and whole grains—no statistically significant differences were observed across the four residential‐SES subgroups. Supporting Information **Table** **A2** presents the estimates for all dietary components, stratified by residential setting and SES across each trimester.

## DISCUSSION

Our main findings indicate that modest differences exist in the intake of certain food groups and nutrients, with rural pregnant participants consuming significantly fewer vegetables in the first trimester, more added sugars from SSBs in early and mid‐pregnancy, and less fiber throughout pregnancy compared to urban pregnant participants. Further, high SES was not only independently associated with a more favorable dietary intake profile but also appeared to modify rural–urban disparities. Notably, pregnant women with low SES, regardless of residential setting, exhibited the least favorable dietary pattern, with disparities particularly pronounced and consistent for added sugars from SSBs and fiber across trimesters when compared to their high‐SES urban counterparts. Another notable finding was the consistency of dietary components within each group across trimesters, challenging assumptions about spontaneous dietary improvement during pregnancy.

The lower intake of vegetables during the first trimester and the reduced fiber consumption across pregnancy among rural participants raise public health concerns, as vegetables are essential sources of vitamins and minerals vital for maternal and fetal health.^24^ Fiber plays a critical role in modulating the gut microbiota, supporting metabolic regulation, and potentially preventing excessive gestational weight gain.^25^, ^26^ Although consistent rural–urban differences were not observed in isolated fiber sources such as fruits and whole grains, the slightly higher overall fiber intake among urban women likely reflects a more favorable diet profile compared to rural women.

In parallel, the consistently higher intake of SSBs among rural participants warrants attention. Excessive consumption of added sugars has been associated with adverse pregnancy outcomes, including increased risk of gestational diabetes, excessive fetal growth, and long‐term metabolic dysfunction.^27^, ^28^ SSBs often reflect broader Western dietary patterns characterized by frequent consumption of ultra‐processed foods.^29^ Such beverages are heavily marketed and readily available, making them common sources of discretionary calories, particularly among socioeconomically disadvantaged groups.^30^

This finding aligns with previous international studies conducted in diverse contexts, which have reported lower diet quality among rural pregnant women. Suliga,^13^ for instance, observed that rural women in Poland consumed fewer vegetables, dairy products, whole grains, and fruit juice than their urban counterparts. In China, Gao et al.^14^ compared pregnant women from rural and urban areas in the Sichuan Province and found significantly lower intakes of key micronutrients, including vitamin A, zinc, iron, calcium, and riboflavin among rural women. By documenting similar disparities by rurality during pregnancy in a US sample, the present study reinforces the global relevance of geographic inequities in maternal nutrition.

These findings regarding rural–urban disparities in maternal diet may be due in part to differences in physical access to healthy foods. Structural barriers in rural areas, such as the limited availability of grocery stores offering a variety of fresh produce and whole‐grain products and a greater reliance on small convenience stores or gas stations as primary food sources, might contribute in part to these differences.^31^ Despite filling this gap, dollar stores and convenience stores typically offer highly processed and less nutrient‐dense foods, which may further limit healthy dietary options in rural communities.^32^

This reasoning is supported by a qualitative study conducted among rural low‐income residents of Minnesota and Iowa finding that, although personal factors influence eating behavior, environmental factors such as limited number of grocery stores, lack of local competition, higher prices, and poor quality and limited variety of food options (both in grocery stores and restaurants) were recurring themes related to food access among participants.^33^ Although not directly assessed in this study, cultural and social norms, as well as taste preferences, may also shape dietary intake in rural areas,^34^ and the complex interplay between access, affordability, and cultural factors warrants further investigation.

Economic constraints can limit access to healthy foods, particularly in rural areas where such items, even when available, are often more expensive than in urban settings. A prospective study among Australian women clearly illustrated these disparities, where pregnant women with higher incomes showed more pronounced improvements in overall diet quality across pregnancy, as measured by the Healthy Eating Index (HEI), compared to their lower‐income counterparts.^35^ Similarly, participants with higher educational attainment showed more pronounced improvements in overall diet quality than those with lower levels of education.^35^ Comparable findings have been observed in the United States. An observational cohort study conducted in North Carolina assessed dietary quality among 383 pregnant women and found significantly lower HEI scores among those with a lower income‐to‐poverty ratio.^36^ Additionally, women without a college education had poorer diet quality, further underscoring the impact of financial constraints on dietary quality during pregnancy.^36^

The intersectional analysis of rural/urban residence and SES indicators highlights how these factors interact to create unique dietary challenges for pregnant women. Our findings indicate that SES exacerbates dietary disparities when comparing extreme ends of the rural/urban and SES spectrums; for instance, urban high‐SES women often had markedly better dietary profiles for fiber, added sugars, and calcium compared to rural low‐SES women. Nevertheless, some of the challenges faced by low‐income women appeared similar regardless of whether they resided in a rural or urban area, as intakes did not differ significantly between these two low‐SES groups. Together, these findings suggest that the combination of rurality and low SES may have a compounding effect on dietary inequities, underscoring the need to explore innovative approaches that address barriers extending beyond the effects of either factor in isolation.

The current findings among pregnant women align with national disparities observed in the general US adult population. A large cohort study^37^ found that adults residing in rural areas had a 61% higher risk of poor diet quality (using the American Cancer Society Diet Score), including lower vegetable intake and higher consumption of SSBs compared to those in metropolitan areas, even after adjusting for SES and other sociodemographic factors. Additionally, even among participants with higher education, living outside metropolitan regions was associated with reduced dietary quality,^37^ suggesting that structural and geographic barriers persist despite individual‐level resources.

The main strength of the study is its prospective cohort design, which included repeated dietary assessments conducted in each trimester of pregnancy, allowing for the observation of changes over time. Data were collected across three distinct geographic sites, which supports the broader relevance of the findings. The study also employed a multidimensional measure of SES and incorporated an intersectional analysis to examine how SES and rurality interact, providing additional insight into patterns of dietary disparities.

However, certain limitations should be acknowledged. The observational nature of the study precludes causal inferences. Although the statistical approach employed handles sample size imbalances appropriately and the sensitivity analyses confirmed the consistency of the findings, a larger rural sample could provide more precise confidence intervals and additional insights not captured in the current analysis. The dichotomization of RUCA codes to define rurality and of the SES score to classify socioeconomic status, while practical for analytical purposes, oversimplifies complex constructs and may result in information loss. Moreover, the study sample was relatively well‐educated, insured, and not racially diverse, which may limit the generalizability of the findings to broader populations of pregnant women in rural and urban areas of the United States or other settings. Dietary intake was assessed using the self‐administered DSQ, a validated screening tool designed to estimate usual intake at the population level. While less precise than repeated 24‐h dietary recalls and subject to recall and social desirability bias, the DSQ offers several advantages, including brevity and low participant burden, making it suitable for longitudinal studies involving multiple assessments.

Future research should employ more comprehensive dietary assessment methods, such as repeated 24‐h recalls, to capture greater dietary variability and validate these findings. Additionally, qualitative studies exploring the lived experiences of pregnant women across different geographic and socioeconomic contexts could inform more targeted and culturally appropriate interventions. Beyond research, policy and programmatic strategies are also needed to reduce disparities linked to rurality and SES. Strategies such as SSB taxation,^38^ subsidies,^39^ and culturally appropriate messaging^40^ can help promote the affordability and accessibility of healthy foods.

## CONCLUSION

This study reveals modest but consistent dietary differences between pregnant women in rural and urban areas, particularly concerning added sugars from SSBs and fiber. While disparities were most pronounced when comparing high‐SES urban women to their low‐SES rural counterparts, the shared nutritional challenges among all low‐SES women reinforce the need for public health strategies that address common structural barriers to healthy eating, targeting this vulnerable population regardless of their location.

## CONFLICT OF INTEREST STATEMENT

The authors declare no conflicts of interest.

## Supporting information



Supporting information
